# Organ-Differentiated Metabolomic Profiling Reveals Putative Associations Between Predicted Bioactivity and Database-Annotated Flavor in Medicinal–Edible *Talinum paniculatum* (Jacq.) Gaertn

**DOI:** 10.3390/metabo16100726

**Published:** 2026-09-29

**Authors:** Haifeng Wang, Da Li, Haoran Xu, Shuyao Wang, Jihao Zhu, Shuangxiang Xu, Jianfeng Dai, Yonghua Zhang, Xinjie Jin, Wanbo Zhang

**Affiliations:** 1 College of Life and Environmental Science, Wenzhou University, Wenzhou 325035, China; 2 Kaihua Yiyun Family Farm, Kaihua, Quzhou 324306, China

**Keywords:** *Talinum paniculatum*, untargeted metabolomics, organ specificity, bioactive compounds, flavor metabolites

## Abstract

Background: Medicinal–edible plants are abundant sources of natural bioactives and functional food precursors. *Talinum paniculatum* (Jacq.) Gaertn is a commonly utilized folk herb, but there is still a lack of systematic metabolomic research linking its organ-specific phytochemical variations to its medicinal and sensory attributes. Methodology: In this work, untargeted UPLC-MS/MS metabolomics was applied to methanolic extracts of the roots, stems, leaves and fruits of *T. paniculatum*. Multivariate statistics and KEGG enrichment screened differentially accumulated metabolites (DAMs), while multi-database matching against TCMSP, FlavorDB, and MFood-Flavor Innovation was performed to prioritize candidates with database-assigned pharmacological or flavor-related annotations. For candidates carrying flavor descriptors, relative abundance values were compared across organs to characterize organ-dependent distribution patterns. Results: After quality control filtering and annotation-based deduplication, 4521 putatively annotated metabolite entries spanning 21 chemical classes were retained across the four organs. Amino acid derivatives constituted the largest class, accounting for 31.72% of all annotated entries. Among the presumptively annotated DAMs, five were further proposed as candidate organ-discriminatory metabolites. TCMSP screening matched 348 metabolites with predicted pharmacological relevance; 55 met the OB and DL screening criteria, and of these, 22 showed preferential accumulation in fruits. Subsequent database annotation highlighted candidates carrying both predicted pharmacological relevance and database-assigned flavor attributes, including umami-associated pyroglutamic acid, sweet neoastilbin, and bitter-tasting melilotoside, helenalin, quercetin, cianidanol and glycitein. These dual-functional metabolites exhibited distinct organ-specific distribution patterns. Conclusions: The organ partitioned distribution of metabolites with database-assigned flavor attributes and predicted bioactivity provides testable hypotheses for selective organ utilization, pending targeted quantification, sensory evaluation, and bioactivity validation.

## 1. Introduction

Medicinal and edible plants possess dual economic value as daily dietary ingredients and traditional medicinal raw materials, making them pivotal resources for developing novel functional foods and natural pharmaceuticals [1,2]. The differential accumulation of phytochemicals across roots, stems, leaves and fruits leads to organ-specific functions, laying a material foundation for the classified utilization of plant organs in food and pharmaceutical industries [3,4]. Untargeted metabolomics coupled with multi-database annotation has become a robust analytical strategy for dissecting the global phytochemical landscape of medicinal–edible plants. Representative studies on *Euchresta japonica*, *Rehmannia chingii*, and *Codonopsis radix* have validated its reliability for identifying organ-distributed bioactive and taste-related metabolites at the whole-metabolome scale [5,6,7]. This approach has also been successfully applied to reveal organ-specific and environmentally regulated secondary metabolism in other medicinal herbs, such as *Andrographis paniculata* [8]. Nevertheless, most existing studies have paid more attention to bioactive ingredients while ignoring taste and umami-related metabolites, and systematic comparative metabolomics across multiple organs remains scarce for most medicinal and edible plants.

*Talinum paniculatum* (Jacq.) Gaertn, commonly known as native ginseng, is a perennial succulent herb distinguished by its tuberous roots, fleshy elliptic to obovate leaves, terminal pink-flowered panicles, and small globose capsules. Originating from tropical and subtropical America, this species has been introduced and widely cultivated across Asia, including in China, as both a medicinal herb and an edible leafy vegetable [9,10]. Ethnobotanical surveys in coastal southern China state that its tender stems and leaves are consumed by stir-frying, boiling or soup-making, while mature roots are often stewed with meat. Its roots are also employed in local folk therapy as a tonic and for the relief of cough and hemoptysis [11,12]. Young *T. paniculatum* foliage is also eaten raw or cooked in Indonesia, while in Brazilian traditional medicine, the plant is utilized to ease gastrointestinal disorders and treat cutaneous wounds and infections, with additional folk uses linked to cardiovascular health [13,14,15].

Previous studies identified chlorogenic acids, amino acid derivatives, nucleosides, O-glycosylated flavones, and organic acids in leaf extracts, together with steroids, saponins, tannins, and triterpenes in diverse extract fractions [13,14]. A recent study identified and quantified 22 metabolites in root extracts, further expanding the known chemical repertoire of its medicinal roots [16]. Available pre-clinical evidence shows that leaf extracts possess in vitro antioxidant and antibacterial activities [11,14,16]. Rodent-model studies have reported diuretic, cardiorenal, and cardioprotective effects, and leaf-derived fractions display gastroprotective activity in mice [14,17,18,19]. However, despite these valuable findings, important knowledge gaps remain. A comprehensive multi-organ metabolomic survey of *T. paniculatum* is still lacking, and organ-specific accumulation patterns of primary and secondary metabolites are poorly resolved. Furthermore, most studies have evaluated pharmacological performance or nutritional properties in isolation. Few analyses integrate bioactive constituents and flavor-related metabolites to systematically interpret its dual medicinal–edible potential. Moreover, the organ-level distribution and putative functions of taste-related compounds remain uncharacterized, restricting targeted high-value utilization of this plant resource.

In this study, we address two research questions: (i) Do roots, stems, leaves, and fruits of *T. paniculatum* differ in their profiles of putatively annotated metabolites? (ii) Can public database annotations help prioritize organ-associated candidate metabolites for subsequent targeted validation? With these questions in mind, untargeted UPLC–MS/MS metabolomics was used to characterize metabolite profiles in roots, stems, leaves, and fruits. Differentially accumulated metabolites (DAMs) were screened, and KEGG pathway enrichment analysis was performed to examine organ-dependent metabolic variation. The TCMSP, FlavorDB, and MFood-Flavor Innovation databases were then queried for predicted pharmacokinetic properties, compound–disease records, and flavor descriptors. These in silico annotations were used only for hypothesis generation, providing a framework for targeted quantification, bioactivity testing, and sensory validation.

## 2. Materials and Methods

### 2.1. Sample Collection and Identification

*T. paniculatum* (Jacq.) Gaertn. were collected in December 2025 from Kaihua County, Quzhou City, Zhejiang Province, China (29.02° N, 118.24° E). Healthy mature individuals at the reproductive developmental stage were selected for metabolomic analysis. Roots, stems, leaves and fruits were separately sampled from three independent plants to serve as biological replicates. All freshly harvested organs were snap-frozen in liquid nitrogen immediately after collection and preserved at −80 °C prior to metabolite extraction. The plant materials were morphologically identified by Prof. Yonghua Zhang based on key diagnostic vegetative and reproductive traits, including succulent stems, obovate fleshy leaves, terminal pink panicles, and small subglobose capsules. These morphological characters were cross-referenced against authoritative taxonomic descriptions of *T. paniculatum*. The iPlant database was utilized solely as an auxiliary digital reference for verification; morphological observation served as the primary basis for species identification.

### 2.2. Sample Preparation and Extraction

Biological samples were vacuum freeze-dried for 63 h using a freeze dryer (Scientz-100F, Ningbo Scientz Biotechnology Co., Ltd., Ningbo, China). The dried samples were then ground into a homogeneous powder at 30 Hz for 1.5 min using a mixer mill (MM 400, Retsch GmbH, Haan, Germany). Exactly 30 mg of powder was accurately weighed using an electronic balance (MS105Dμ, Mettler Toledo, Zurich, Switzerland) and extracted with 1500 μL of 70% (*v*/*v*) methanol aqueous solution pre-cooled at −20 °C and containing internal standard 2-chlorophenylalanine (CAS 103616-89-3, Sigma-Aldrich, St. Louis, MO, USA). The internal standard solution was prepared as follows: 1 mg of reference standard was dissolved in 1 mL of 70% methanol to prepare a 1000 μg/mL stock solution, which was further diluted with 70% methanol to yield a working internal standard solution of 250 μg/mL. During extraction, the mixture was vortexed for 30 s every 30 min, and this procedure was repeated six times to improve extraction efficiency. After centrifugation was performed at 12,000 rpm for 3 min, the supernatant was collected, filtered through a 0.22 μm microporous membrane, and transferred into UPLC vials for subsequent UPLC-MS/MS analysis.

### 2.3. Untargeted Metabolomics Analysis

#### 2.3.1. UPLC-MS/MS Conditions

Analysis was performed on an AB TripleTOF 6600 system (SCIEX, Foster City, CA, USA) coupled with a Waters ACQUITY UPLC system (Waters Corporation, Milford, MA, USA). Separation was carried out using an HSS T3 column (1.8 μm particle size, 2.1 mm internal diameter × 100 mm length) at 40 °C. The mobile phases were 0.1% formic acid in water (A) and 0.1% formic acid in acetonitrile (B). The gradient elution program was set as follows: 0–5 min, 5–65% B; 5–6 min, 65–99% B; 6.0–7.5 min, 99% B; 7.5–7.6 min, 99–5% B; and 7.6–10 min, 5% B. The flow rate was 0.40 mL/min; the injection volume was 4 μL.

ESI^+^ and ESI^−^ modes were applied. The ion spray voltages were 5000 V (ESI^+^) and −4000 V (ESI^−^). The source temperature was 550 °C. The ion source gas 1 measurement was 50 psi, that for gas 2 was 60 psi, and that for curtain gas was 35 psi. The declustering potential values were 80 V (ESI^+^) and −80 V (ESI^−^). For MS^1^, the collision energies were 10 V (ESI^+^) and −10 V (ESI^−^), with a mass range of 50–1250 Da, whereas for MS^2^, the collision energies were 30 V (ESI^+^) and −30 V (ESI^−^), with a mass range of 25–1250 Da.

#### 2.3.2. Data Quality Control and Preprocessing

The UPLC-MS/MS detection was assisted by Wuhan Metware Biotechnology Co., Ltd. (Wuhan, China). QC samples were prepared by pooling equal aliquots of all samples and analyzed every 10 injections. Raw data were converted to mzML and processed by XCMS online (https://xcmsonline.scripps.edu; accessed on 9 January 2026) for peak picking, alignment, and retention time correction [20]. Features with a missing rate > 50% were removed. Missing values were imputed by the KNN algorithm, implemented in R with the impute package (version 1.56.0) using default parameters, or one-fifth of the minimum intensity. Peak intensities were corrected by SVR. The values in Table S1 represent normalized peak areas and indicate relative metabolite abundances. These values were used only to compare the same metabolite across samples or experimental groups. Untargeted metabolomic analysis and metabolite annotation were performed by Metware Biotechnology (Wuhan, China) using its in-house Metware Database (MWDB, version 6.7). Spectral matching scores combined weighted contributions from precursor ion mass error, fragment ion matching, and similarity between experimental and library MS/MS spectra. Annotation confidence was assigned according to the Metabolomics Standards Initiative (MSI) classification system.

Positive- and negative-ion datasets were combined after annotation and quality-control filtering because the two ionization modes provide complementary metabolite coverage. Entries with an integrated annotation score > 0.5 and a QC coefficient of variation < 0.5 were retained. When the same compound identity was annotated more than once, the entry with the higher reported qualitative level was retained; when the qualitative levels were identical, the entry with the higher annotation score was retained. This procedure represents annotation-based deduplication rather than complete compound-level deconvolution. In this study, a detected feature denotes an aligned LC–MS peak defined by its *m*/*z* and retention time after preprocessing, whereas a putatively annotated metabolite denotes a feature assigned a candidate identity through the database and spectral-annotation workflow. A confirmed metabolite requires agreement with an authentic standard in both retention time and MS/MS fragmentation under the same analytical conditions. Product-ion MS/MS data acquired in information-dependent acquisition mode contributed to the putative annotation workflow. Isotope exclusion within 4 Da was applied during candidate-ion selection to reduce redundant MS/MS triggering; however, this acquisition setting did not constitute post-acquisition grouping of isotope peaks, adducts, coeluting features, or in-source fragments. As no metabolite in the present dataset was confirmed using an authentic standard, the final dataset is reported as 4521 annotation-based deduplicated putative entries rather than 4521 confirmed unique chemical structures.

In this study, metabolite assignments were classified into three levels following metabolomics community standards: (i) detected features: aligned LC-MS peaks defined by *m*/*z* and retention time; (ii) putatively annotated metabolites: features assigned candidate identities via spectral library matching, without authentic standard confirmation; and (iii) confirmed metabolites: identities verified by authentic reference standards. All metabolites reported here are putatively annotated; no confirmed metabolites are included.

### 2.4. Differential Metabolite Analysis and Clustering

Three biological replicates per organ were used in this exploratory metabolomics study, consistent with common practice in untargeted plant metabolomics. Intra-group biological variability was assessed via Pearson correlation coefficients and principal component analysis (PCA). Prior to multivariate statistical analysis, the raw metabolomics data were log_2_-transformed and scaled to unit variance (UV). Unsupervised PCA was performed using the prcomp function in R (www.r-project.org) to evaluate the overall metabolic variation among samples and the consistency of biological replicates within each organ. Pearson correlation coefficients (PCCs) between samples were calculated using the cor function in R to further assess within-group similarity and between-sample variation. Heatmaps for both sample and metabolite clustering were generated using the R package Complex Heatmap (version 2.9.4), where hierarchical cluster analysis (HCA) was visualized based on PCC values [21]. For HCA, dendrograms were constructed, and normalized metabolite signal intensities were scaled to unit variance (UV) before being displayed as a color gradient.

To screen for DAMs across groups, orthogonal partial least squares-discriminant analysis (OPLS-DA) was conducted for each pairwise organ comparison using the MetaboAnalystR package (version 1.0.1) following log_2_ transformation and UV scaling [22]. R^2^X and R^2^Y were used to summarize the variation explained in the predictor and response matrices, respectively, whereas Q^2^ was used to evaluate cross-validated predictive performance. To assess potential model overfitting, 200-permutation tests were performed by randomly reassigning group labels and recalculating the OPLS-DA model for each permutation. The permutation analysis recorded the number of randomized models with R^2^Y or Q^2^ values exceeding those of the corresponding original model. The resulting permutation *p* values were used solely to evaluate OPLS-DA model performance and were not used as metabolite-level *p* values from differential testing. Given the limited number of biological replicates (*n* = 3 per organ), OPLS-DA was used primarily for exploratory visualization and variable importance in projection (VIP) ranking rather than as an independently validated classification model. Differentially accumulated metabolites were identified using VIP > 1 and |log_2_ fold change (FC)| ≥ 1. Finally, all differentially expressed metabolites were annotated based on the KEGG Compound database (http://www.kegg.jp/kegg/compound/; accessed on 9 January 2026) and further mapped to the KEGG Pathway database (http://www.kegg.jp/kegg/pathway.html; accessed on 9 January 2026) for functional enrichment analysis [23]. For the six candidates metabolites selected for subsequent analysis, relative abundance values were summarized as the mean ± standard deviation of three biological replicates per organ. Differences between four organs for each metabolite were evaluated using one-way analysis of variance (one-way ANOVA). When the omnibus ANOVA reached significance at *p* < 0.05, Tukey’s honest significant difference (HSD) test was implemented for post hoc pairwise multiple comparisons (*α* = 0.05).

For pairwise-group volcano plot generation of untargeted metabolomics data, a two-tailed Student’s t-test was used to calculate *p* values between two organ groups. DAMs were determined by combining the variable importance in projection (VIP > 1) from OPLS-DA models and fold-change threshold (|log_2_FC| ≥ 1). The permutation test (200 iterations) was only used to evaluate the overfitting risk of OPLS-DA models and not for metabolite level significance calculation.

### 2.5. Screening of Potential Bioactive Components

All presumptively annotated metabolites were mapped to the Traditional Chinese Medicine Systems Pharmacology Database and Analysis Platform (TCMSP, https://tcmsp-e.com; accessed on 9 February 2026) [24]. Potential bioactive compounds were screened based on ADME properties, namely oral bioavailability (OB) ≥ 30% and drug-likeness (DL) ≥ 0.18, according to previously reported criteria for medicinal and edible plants [25]. Compounds matching the same metabolite by name, molecular formula, or InChIKey were retained. Metabolites without matched records in TCMSP were excluded from subsequent bioactivity analysis. Compound–disease records associated with the TCMSP-matched candidates were retrieved directly from TCMSP and its indexed literature. Disease-term frequencies were calculated only as descriptive counts of the retrieved database records. No disease-enrichment analysis, disease-specific bioactivity assay, therapeutic-efficacy evaluation, or clinical-relevance assessment was performed.

### 2.6. Flavor Characterization and Umami Component Identification

To characterize the flavor attributes of *T. paniculatum*, including sweetness, bitterness, fruitiness, greenness, floralness, and spiciness, the flavor properties of all detected metabolites were retrieved from the FlavorDB database (https://cosylab.iiitd.edu.in/flavordb/; accessed on 19 March 2026) and categorized as sweet, bitter, fruity, green, floral, woody, or spicy [26]. For amino acid-related metabolites, InChIKey identifiers were obtained from PubChem (https://pubchem.ncbi.nlm.nih.gov/; accessed on 19 March 2026) and HMDB (https://hmdb.ca/metabolites; accessed on 19 March 2026) using their PubChem CIDs and HMDB IDs. Structural classification was performed via the ClassyFire hierarchical ontology using the classyfireR package (version 0.3.8) [27]. Only metabolites with valid InChIKeys and complete annotations were retained; unannotated features were excluded. The structurally classified metabolites were classified into four categories: peptidomimetics (class level), dipeptides/oligopeptides (level 6), other peptides (level 5), and amino acid derivatives and non-peptide metabolites (monomeric free amino acids and their derivatives). Finally, the subclassified amino acid derivatives were queried against the MFood-Flavor Innovation (MFFI) database (Shanghai Jiao Tong University, https://mffi.sjtu.edu.cn/database; accessed on 19 March 2026) to identify umami-related metabolites. Flavor descriptors were retrieved from FlavorDB and MFood-Flavor Innovation by matching compound names and PubChem identifiers. These descriptors represent previously reported database annotations and were not treated as experimentally measured sensory properties of *T. paniculatum*. The untargeted metabolomic analysis provided peak area-based relative signal intensities but did not determine absolute concentrations, compound-specific sensory recognition thresholds, matrix effects, or sensory responses.

## 3. Results

### 3.1. Identification and Profiling of Metabolites Across the Four Organs

In this study, an untargeted UPLC-MS/MS metabolomics approach was employed to comprehensively identify metabolites in four organs of *T. paniculatum* (Figure 1A). Multiple quality control (QC) assessments were performed to verify the reliability of the metabolomic analysis. The total ion chromatograms (TICs) of all QC samples exhibited high overlap (Figure S1), accompanied by strong Pearson correlation coefficients (Figure S2) and low coefficients of variation (CV) for metabolite quantification (Figure S3). This validation confirmed the excellent stability and reproducibility of the established analytical platform for subsequent metabolite profiling.

After quality-control filtering and removal of redundant annotations, 4521 metabolites were putatively annotated across the four organs of *T. paniculatum* (Table S1). These entries represent database- and MS/MS-supported candidate assignments rather than authentic standard-confirmed metabolite structures. Amino acids and their derivatives made up the dominant chemical group, containing 1434 metabolites and accounting for 31.72% of all annotated substances. Organic acids took second place with 589 metabolites (13.03%), followed by unclassified compounds (520, 11.50%), benzene and its substituted derivatives (465, 10.29%), and alkaloids (226, 5.00%) (Figure 1B). The unclassified metabolites were then sorted into 11 subcategories, among which ketones, lactones and saccharides occupied the highest proportions (Figure 1C). Taken together, amino acid derivatives and low-molecular-weight organics constitute the core metabolic components of *T. paniculatum*. Such compositional characteristics lay a solid foundation for subsequent comparative analysis of metabolite differences among individual organs.

### 3.2. Metabolic Distinctions and Screening of DAMs

To further clarify metabolic variations among different *T. paniculatum* organs, PCA was carried out to visualize organ-specific metabolite features across roots, stems, leaves and fruits (Figure 2A). The top two principal components captured 76.50% of total metabolic variation, with leaf samples fully separated from the remaining three organs, pointing to a potential unique metabolic profile specific to leaves. Figure 2B illustrates the overlap of DAMs derived from the six pairwise organ-comparison analyses. A total of 337 DAMs were shared across all six comparison groups. In this overlap analysis, 63 DAMs exclusively detected only in the stem-versus-root comparison (absent from the other five pairwise comparisons). For the stem-versus-root comparison, the full dataset yielded 2350 DAMs in total, consisting of 1623 upregulated and 727 downregulated metabolites.

OPLS-DA models were validated for every pairwise comparison to mine organ-enriched DAMs (Figure S4). Compounds satisfying VIP > 1 and |log_2_FC| ≥ 1 were regarded as DAMs. All qualifying DAMs were then grouped via *K*-means clustering into eight separate subclusters (Figure 2C). This grouping clearly illustrated how metabolite abundance shifts with organ type, and offers chemical evidence to support targeted exploitation of different plant parts. Cluster data revealed widespread organ-biased metabolite accumulation. Subcluster 6 (1260 metabolites) and Subcluster 1 (1116 metabolites) constituted the two largest metabolite cohorts, accumulating predominantly in leaves and fruits, respectively. The eight clusters fell into two broad categories: metabolites enriched in only one organ, and those abundant across two organs. Subclusters 5, 6, 8 and 1 featured single-organ enrichment for roots, leaves, stems and fruits in sequence. Other clusters showed dual-organ distribution: Subcluster 3 concentrated in roots and leaves, Subcluster 2 in roots and fruits, Subcluster 7 in roots and stems, and Subcluster 4 in leaves and fruits.

Furthermore, hierarchical clustering of the full metabolite dataset was also performed to cross-check global metabolic dissimilarities across organs (Figure 2D). Fruits and leaves each formed isolated branches, showcasing the divergent metabolic landscapes. Distinct blocks of enriched signals within the heatmap directly reflect organ-restricted metabolite distribution. Such uneven chemical partitioning may confer distinct metabolic traits and divergent functional potential to each organ of *T. paniculatum*.

### 3.3. Screening and KEGG Enrichments of Candidate Discriminatory Metabolites

To further prioritize candidate metabolites with robust organ-discriminatory power, we analyzed the top-ranked DAMs from all six pairwise comparisons (Figure 3A–F). Volcano plots and VIP rankings visualized the overall differential patterns and highlighted metabolites with strong discriminatory capacity in each comparison. The most obvious metabolic divergence occurred between leaves and roots, where a total of 2892 DAMs were annotated, consisting of 2019 upregulated and 873 downregulated compounds. The stem-versus-root comparison yielded 2350 DAMs, the fewest among the six pairwise comparisons. Of these, 1623 were upregulated and 727 were downregulated. The top 20 metabolites with the highest VIP values were further extracted, and were regarded as pivotal substances driving metabolic differentiation among different organs.

To prioritize organ-discriminatory candidates from the large DAM pool, we adopted a recurrence-based filtering strategy. This approach prioritizes metabolites with consistently high VIP rankings across multiple independent pairwise comparisons, which reduces selection bias from comparison-specific outliers and improves the robustness of candidate prioritization in exploratory untargeted metabolomics [3]. Specifically, the top 20 VIP-ranked metabolites from each of the six OPLS-DA comparisons were pooled and deduplicated, yielding 85 unique DAMs, 29 of which appeared in ≥ 2 top-20 lists. Further filtering for recurrence in ≥ 3 pairwise comparisons, combined with the DAM thresholds (VIP > 1, |log_2_FC| ≥ 1), yielded five candidate organ-discriminatory metabolites: cabucine, (2-oxo-2,3-dihydro-1H-indol-3-yl) acetic acid, 3,4-dihydroxybenzaldehyde, delphinidin 3-(6′’-malonylglucoside) 5-glucoside, and *β*-lactose. These span alkaloids, phenolic acids, flavonoids, and carbohydrates, respectively (Figure 3G). Cabucine and 3,4-dihydroxybenzaldehyde were primarily associated with fruit-related comparisons, while (2-oxo-2,3-dihydro-1H-indol-3-yl) acetic acid appeared in four pairwise comparisons, suggesting a broad role in inter-organ metabolic differentiation. The remaining two metabolites each recurred in three comparisons. These five metabolites represent high-priority targets for subsequent structural confirmation and functional validation. Notably, this recurrence-based ranking is intended solely for candidate prioritization and does not constitute validated biomarkers.

KEGG pathway enrichment analysis was performed to characterize the functional signatures of DAMs across all six pairwise comparisons (Figure 4A–F). ABC transporters (ko02010) was the most prominently enriched pathway across multiple groups, and dominated the stem vs. root, fruit vs. leaf, and leaf vs. root comparisons. This pattern aligns with the known role of ABC transporters in mediating inter-organ allocation of phytochemicals and nutrients, providing a tentative mechanistic basis for the observed organ-specific phytochemical accumulation [3,4,5]. Nucleotide metabolism (ko012320) and purine metabolism (ko00230) were significantly enriched in fruit-related comparisons, consistent with active cell proliferation and nutrient reserve accumulation in fruits as reproductive organs [4,28]. Amino sugar and nucleotide sugar metabolism (ko00520) was most strongly enriched in the stem vs. root comparison, corresponding to structural polysaccharide and cell wall metabolism during vegetative organ growth [3,29]. Notably, glucosinolate biosynthesis (ko00966) was differentially enriched in the stem vs. leaf comparison. Given that glucosinolates are characteristic secondary metabolites of Brassicaceae and have not been reported in Talinaceae, this enrichment signal most likely reflects the automated mapping of other sulfur-containing glycosides onto this KEGG category, rather than the presence of an intact glucosinolate biosynthetic pathway in *T. paniculatum* [3,30]. Nevertheless, this result indicates divergent sulfur-containing secondary metabolism between vegetative organs, which may be associated with stress defense and leaf taste properties. Amino acid metabolism pathways were broadly enriched across all comparisons. Core amino acid biosynthesis (ko01230) was highly enriched in vegetative organ comparisons, consistent with active nitrogen assimilation in leaves and the role of amino acids as precursors for defensive and medicinal secondary metabolites in roots and stems [9,31]. Valine, leucine and isoleucine degradation (ko00280) and histidine metabolism (ko00340) were also enriched in the stem vs. root comparison. This widespread enrichment of amino acid-related pathways is consistent with the high abundance of amino acid derivatives detected, and reflects active synthesis and inter-organ distribution to support organ growth and functional differentiation.

### 3.4. TCMSP-Based Prioritization of Candidate Metabolites with Putative Bioactivity

To assess metabolites with putative pharmacological relevance in *T. paniculatum*, all 4521 annotated metabolites were subjected to in silico screening using the TCMSP database. We systematically analyzed the medicinal annotations, chemical classification, and organ-specific accumulation patterns of candidate compounds, together with filtering based on OB and DL parameters.

A total of 348 putatively annotated metabolites were successfully matched to entries in the TCMSP database (Figure 5A, Table S3). These compounds covered diverse chemical classes, including 58 phenolic acids, 45 alkaloids, and 41 flavonoids, as well as terpenoids, benzene derivatives, heterocyclic compounds, and other phytochemical subgroups. To prioritize potential candidate compounds for further investigation, we applied the commonly used thresholds of oral bioavailability (OB) ≥ 30% and drug-likeness (DL) ≥ 0.18. Of the 348 matched entries, 55 met both prioritization criteria, with flavonoids (14 entries) and alkaloids (13 entries) representing the two largest groups (Figure 5B). The disease terms summarized in Figure 5C and Table S4 were inherited from the TCMSP compound–disease records. Figure 5C reports the frequency with which these disease terms occurred among the database records associated with the matched candidates. These frequencies are descriptive properties of the TCMSP database records and should not be interpreted as indicative of disease enrichment, experimentally demonstrated pharmacological activity, therapeutic efficacy, or clinical relevance of *T. paniculatum*.

TCMSP aggregates disease–metabolite associations curated from the published literature [24]. We summarized frequently reported disease entries and corresponding candidate metabolites (Figure 5C,D, Table S4). Quercetin displayed the greatest number of disease-related annotations, consistent with broad pharmacological evidence reported in previous studies [25,26,27,32,33]. The heatmap based on relative abundance further uncovered distinct organ-specific accumulation patterns for these 55 candidates (Figure 5E). Among the 55 OB- and DL-screened candidates, 22 showed preferential accumulation in fruits, the largest number among the four organs. Among all candidates, cimigenol and triptophenolide exhibited the highest overall abundance across the four organs.

### 3.5. Identification of Organ-Specific Flavor-Related Metabolic Patterns

Based on global metabolite profiles obtained from untargeted metabolomics, we explored flavor-associated chemical features across organs of *T. paniculatum* to characterize metabolic patterns underlying its edible quality. All annotated compounds were first subjected to structural classification using the ClassyFire database. In total, 895 metabolites obtained definitive structural classification and were assigned to five subgroups: amino acids and non-peptide compounds (409, 45.70%), other peptides (223, 24.92%), oligopeptides (105, 11.73%), dipeptides (96, 10.73%), and peptidomimetics (62, 6.93%) (Figure 6A). To identify candidate compounds potentially linked to umami taste, the refined dataset of amino acids and peptides was further annotated against the MFFI database. Eighteen metabolites received putative umami annotations from MFFI, with the majority belonging to peptide-related classes (Figure 6B, Table S6), comprising eleven dipeptides, four oligopeptides, two other peptides, and one non-peptide amino acid. Notably, Thr-Glu and Glu-Thr were annotated as separate putative sequence-isomeric dipeptides based on distinct retention times and spectral matching. Although they are chemically distinct due to their reversed amino acid sequences, their exact sequence assignments remain provisional without authentic standard confirmation. These database-annotated umami candidates showed organ-specific relative signal patterns, with several candidates exhibiting higher signals in fruits (Figure 6C). Among these umami-related candidates, Glu-Leu and Glu-Val exhibited comparatively high cumulative abundance across the four organs. Only pyroglutamic acid had TCMSP-derived OB and DL values, marking it as a candidate with both an MFFI-derived umami annotation and predicted pharmacological relevance.

Subsequently, compound information was mapped to the FlavorDB database using chemical names and PubChem identifiers to retrieve previously reported flavor associations. In total, 208 metabolites were assigned flavor annotation labels and categorized into seven classes: sweet, bitter, fruity, green, floral, woody, and spicy (Table S7). Sweet- and bitter-annotated metabolites constituted the two largest fractions, accounting for 42.40% and 34.90% of all flavor-annotated metabolites, respectively (Figure 6D). The heatmap showed that sweet-labeled candidates had higher relative signal intensities in fruits, while bitter-annotated compounds were more abundant in leaves (Figure 6E,F). These computationally derived patterns only reflect organ-specific distributions of compounds with previously reported flavor associations. Notably, metabolites present at trace levels below their compound-specific sensory recognition thresholds do not contribute to perceptible taste. Since absolute metabolite concentrations and compound-specific sensory recognition thresholds were not determined in this untargeted metabolomics study, we cannot assess whether individual compounds are present at levels sufficient to contribute to actual taste or aroma perception. Accordingly, these results provide a candidate set of flavor-associated metabolites for follow-up investigation, rather than evidence of the actual sensory properties or palatability of *T. paniculatum* organs. Targeted quantification and formal sensory evaluation are required to validate these in silico predictions.

### 3.6. Identification and Evaluation of Dual-Functional Metabolites with Both Medicinal and Flavor Properties

To further explore the chemical basis underlying the dual medicinal and edible characteristics of *T. paniculatum*, we screened candidate dual-functional metabolites that possessed both predicted pharmacological relevance and database-annotated flavor traits. Compounds were filtered by TCMSP pharmacokinetic criteria (OB ≥ 30%, DL ≥ 0.18) together with FlavorDB flavor annotations. Six metabolites were retained after screening: neoastilbin, quercetin, cianidanol, glycitein, melilotoside, and helenalin. Four of these candidates are flavonoids, melilotoside is classified as a benzene derivative, and helenalin is a heterocyclic compound (Figure 7A–C).

Database-derived flavor annotations suggested distinct taste features among these candidates. Neoastilbin was described as sweet-tasting, whereas the remaining five metabolites were assigned bitter-taste attributes. These in silico observations imply a tentative association between bitter secondary metabolites and the putative medicinal potential, and highlight flavonoids as key candidate compounds contributing to its dual medicinal–edible properties. Further spatial distribution analysis revealed obvious organ-specific accumulation patterns for these dual-functional candidates. The bitter heterocyclic compound, helenalin, predominantly accumulated in roots, showing markedly higher abundance relative to other organs (Figure 7B, Table S1). The bitter flavonoid, glycitein, exhibited preferential accumulation in leaves (Figure 7C). The only sweet-annotated candidate in this dataset, neoastilbin, was highly enriched in fruits with abundance dozens-fold higher than in roots, stems and leaves. Melilotoside and quercetin also reached their maximum abundance in fruits (Figure 7). In contrast, cianidanol showed stem-preferential distribution, though its overall abundance was relatively low compared with the other candidate metabolites.

## 4. Discussion

### 4.1. An Organ-Resolved Metabolomic View Expands Current Knowledge of T. paniculatum

In this study, untargeted UPLC-MS/MS metabolomics was applied to systematically compare metabolite profiles across the roots, stems, leaves, and fruits of *T. paniculatum*. Previous pharmacological and physiological studies have documented the nutritional, antimicrobial, cardiorenal, cardioprotective, and gastroprotective properties of *T. paniculatum* extracts based on in vitro assays and animal models [14,15,17,18,30]. However, these studies did not clarify the identities of the underlying metabolites or further delineate their potential functional activities. Collectively, our study constructs a comprehensive organ-resolved chemical landscape of *T. paniculatum*, providing a prioritized candidate pool for subsequent targeted metabolite verification and functional validation.

Building on the presumptively annotated metabolite profiling, the comparisons of roots, stems, leaves and fruits further suggest that organ type may serve as a key factor shaping metabolic characteristics in *T. paniculatum*. Multivariate analyses showed that leaves were most clearly separated from the other organs and contained the largest cluster of metabolites. In addition, fruits also showed a distinct metabolic profile with higher accumulation of sweet-associated metabolites, umami-associated peptides, and several TCMSP-matched candidates [28]. In contrast, roots and stems showed lower organ-preferential patterns, as reflected by the enrichment of helenalin in roots and cianidanol in stems (Figure 7B,C). Notably, leaves and fruits exhibited the most divergent metabolomic profiles, accompanied by higher relative abundance of FlavorDB-, MFFI-, and TCMSP-annotated candidate metabolites. Thus, metabolite allocation in *T. paniculatum* presents strong organ specificity: leaves tend to accumulate primary metabolites and defensive specialized compounds; fruits preferentially enrich metabolites with predicted edible sensory qualities and dual medicinal–flavor properties; while roots and stems synthesize a narrower spectrum of metabolites putatively associated with medicinal functions and structural growth [5,28].

This organ-partitioned metabolic pattern is consistent with findings from previous metabolomic investigations of medicinal and edible plants. Organ-specific metabolite profiling of *E. japonica* has linked organ-resolved chemical variation to plant health-promoting potential, and comparative metabolomics of *R. chingii* revealed distinct organ distribution of predicted bioactive compounds [5,6]. Similarly, metabolite comparison among *Codonopsis* species has clarified chemical consistency and their potential medicinal value [7]. Broadly, fruits are widely recognized as key sites of phytochemical diversification in medicinal plants [4]. In line with these reports, our results reveal a unique chemical feature of *T. paniculatum*: fruits are chemically distinct from leaves and enriched in sweet, umami and putative bioactive metabolites, whereas leaves accumulate more bitter specialized metabolites. This contrast highlights the necessity of integrating flavor annotation and predictive bioactivity information to understand the chemical basis of dual-purpose medicinal–edible plants.

At the chemical class level, the detection of flavonoids, phenolic acids, alkaloids, and saponins is consistent with previous phytochemical and pharmacological investigations of *T. paniculatum* and related species [14,15,16,17,18]. Differing from prior studies, the present study presumptively annotated amino acid derivatives as the dominant metabolite group, updating the current understanding of the plant’s chemical composition. This result indicates that the sensory, nutritional, and functional potential of *T. paniculatum* may rely not only on classic phenolic and flavonoid components, but also on a complex nitrogen-containing metabolic network [31]. The organ-resolved metabolomic dataset provides a systematic chemical resource to guide follow-up targeted quantification, sensory validation, and bioactivity assays. Based on this overall metabolic characteristic, we further explored the compositional features and putative functions of the dominant amino acid derivatives in *T. paniculatum*.

### 4.2. Amino Acid Derivatives May Shape Edible Quality and Potential Germplasm Evaluation Indicators

A total of 1434 amino acid-derived metabolites were putatively annotated in *T. paniculatum*; of these, 895 were further categorized into free amino acids, dipeptides, oligopeptides, peptidomimetics, and other peptide subgroups. It should be noted that definitive structural confirmation of these compounds requires verification using authentic standards. Even so, our metabolomic data revealed substantially higher abundance and structural diversity of peptide-related constituents in *T. paniculatum* than previously reported, expanding the current understanding of its nitrogenous chemical composition.

Amino acids and small peptides are established determinants of sensory quality in edible plants [34,35,36,37], and modern flavor research increasingly integrates peptidomic profiling with bioinformatic annotation [36,37,38]. Consistent with previous studies on edible plant and fungal materials, amino acid derivatives and peptide metabolites in *T. paniculatum* were predicted to contribute to its taste characteristics [39,40]. In particular, fruit-enriched metabolites annotated via the MFFI database provide preliminary candidate compounds that may underlie the organ-specific sensory traits of *T. paniculatum*. The co-occurrence of diverse flavor-related metabolites and putative bioactive compounds helps explain the dual medicinal and edible value of this species (Figure 5 and Figure 6). Furthermore, the distinct organ-associated distribution patterns of flavor-related metabolites may inform the future evaluation and utilization of different *T. paniculatum* organs. Given their wide distribution, high diversity, and organ-associated accumulation patterns, amino acid and peptide derivatives may also serve as tentative candidate indicators for germplasm screening and flavor-oriented breeding.

Beyond metabolite profiling, however, comprehensive evaluation of plant germplasm can benefit from the integration of phenotypic, molecular, and cytological evidence. For example, Hu et al. combined microspore culture, chromosome-specific PCR, fluorescence in situ hybridization, and phenotypic and cytological analyses to characterize *Brassica napus–Raphanus sativus* disomic alien addition lines [41]. Although these approaches address a different biological level from metabolomics, they illustrate the value of complementary evidence in plant-germplasm characterization. Future studies of *T. paniculatum* should therefore integrate targeted metabolite validation with broader genetic, cytological, and phenotypic characterization across diverse germplasm resources.

### 4.3. Leaves Represent Metabolic Specialization, While Fruits Exhibit Superior Edible and Functional Potential

Multivariate analysis and differentially accumulated metabolite profiling provided further confirmation of distinct specific metabolic signatures across the *T. paniculatum* organs. Leaves displayed the most divergent metabolic profile, especially relative to roots, which aligns with their core physiological functions in photosynthesis and environmental stress adaptation that rely on specialized metabolic reprogramming [29]. The enrichment of amino acid metabolic pathways in leaves also indicates their potential involvement in nitrogen turnover and metabolic homeostasis, a conserved metabolic trait observed in other medicinal plants including *E. japonica* and *R. chingii* [5,6]. In addition, leaves accumulated abundant database-annotated bitter metabolites and high levels of flavonoids, which preliminarily suggests their potential as candidate materials for antioxidant screening, pending further experimental verification. In comparison, fruits presented unique metabolic characteristics linked to putative edible and functional potential. As reproductive organs, fruits commonly accumulate nutrients, flavor-related metabolites and specialized compounds to support seed development and dispersal [17,28]. In this study, the fruits of *T. paniculatum* were specifically enriched in metabolites with FlavorDB- and MFFI-annotated sweet and umami traits, providing a hypothetical chemical basis for their traditional edible value. Therefore, the metabolic differentiation across organs could reflect distinct metabolic strategies: leaves prioritize the accumulation of specialized metabolites putatively associated with defense and stress tolerance, while fruits tend to enrich sensory-active and dual-functional candidate metabolites, laying a theoretical foundation for the organ-specific utilization of *T. paniculatum*.

Crucially, all sensory and functional interpretations are derived exclusively from untargeted metabolomic profiling and public database annotation, without supporting experimental validation. These organ-specific metabolic features therefore represent testable hypotheses rather than conclusive evidence of organ-specific bioactivity or palatability [3,30]. Further screening of flavor and bioactive candidates identified pyroglutamic acid as the only umami-related metabolite that meets the TCMSP-based OB and DL screening criteria (Figure 6C). This fruit-enriched amino acid derivative has been reported to regulate systemic metabolism and functional enzyme activity in other biological systems [42,43], supporting its candidacy as a dual-functional metabolite in *T. paniculatum*. Nevertheless, these literature-reported properties cannot confirm its actual sensory performance or bioactivity in *T. paniculatum* fruits. Targeted functional and sensory validation is still required to clarify its in planta physiological role.

### 4.4. Organ-Specific Enrichment of Bioactive Metabolites Supports Targeted Utilization Strategies

ADME-based screening is a widely adopted in silico strategy that prioritizes phytochemical candidates with predicted pharmacological feasibility, enabling effective narrowing of metabolite pools for further functional exploration [24,44]. In this study, TCMSP-based ADME filtering retained 55 candidate metabolites, mainly flavonoids and alkaloids. Database disease annotations indicated that these candidates are potentially associated with tumor, inflammatory, neurodegenerative, and cardiovascular pathways, which is broadly consistent with the previously reported pharmacological properties of *T. paniculatum* extracts [24,30].

Metabolomic profiling further revealed prominent organ-specific accumulation patterns among these ADME-screened candidates. Most compounds exhibited divergent organ-specific distribution patterns, providing a chemical basis for future targeted compound isolation and experimental validation (Table S1). Several representative metabolites showed clear organ tropism: helenalin predominantly accumulated in roots; glycitein was enriched in leaves; quercetin, neoastilbin, and melilotoside were highly abundant in fruits; and cianidanol primarily accumulated in stems. Consistent with the well-documented antioxidant and anti-inflammatory activities of quercetin [45,46], its preferential accumulation in fruits provides preliminary support for the putative medicinal potential of *T. paniculatum* fruits, and highlights quercetin as a high-priority candidate for follow-up investigation. However, definitive confirmation of its absolute abundance, in planta bioactivity, and functional role requires targeted quantification and experimental validation. However, such functional inferences are based on evidence from the literature and metabolic profiling characteristics, and the actual bioactivity of quercetin within *T. paniculatum* organs still requires further targeted experimental validation. Additionally, neoastilbin was the only candidate concurrently annotated with putative pharmacological relevance (TCMSP) and sweet flavor traits (FlavorDB). This dual database annotation supports prioritization of neoastilbin for testing possible links between flavor characteristics and pharmacological activity in fruits. Nevertheless, all sensory and functional attributes described herein are purely in silico predictions and lack experimental verification. Accordingly, neoastilbin should be regarded as a high-priority candidate for future sensory, pharmacological, and safety assessment, rather than a validated dual-functional compound.

Overall, the organ-biased relative-signal patterns of these candidate metabolites provide a basis for prioritizing compounds and organs for further targeted investigation, rather than establishing organ superiority or suitability for therapeutic or food applications. The comparatively strong root-associated signal for the putative helenalin annotation may justify further investigation of roots in relation to anti-inflammatory constituents and the traditional medicinal use of *T. paniculatum*. Similarly, the flavonoid-associated patterns observed in leaves identify them as candidates for further screening of potentially antioxidant and anti-inflammatory compounds, while the occurrence of multiple flavor-related and ADME-qualified annotations in fruits supports their further evaluation in the context of functional-food development. However, these interpretations remain preliminary. Helenalin, flavonoid, and flavor-related annotations require compound confirmation, absolute quantification, safety assessment, and relevant functional or sensory assays before any conclusions regarding biological activity, organ-specific advantages, or utilization potential can be drawn.

### 4.5. Integrating Flavor and Bioactivity Data to Establish a Comprehensive Assessment System for Medicinal–Edible Herbs

Six metabolites carrying both flavor annotations and predicted bioactivity were screened. Combining flavor-related annotations and predicted bioactivity information enables a holistic interpretation of the dual medicinal–edible properties of *T. paniculatum*. Sweet-tasting candidate metabolites preferentially accumulate in fruits, whereas bitter-annotated compounds are abundant in leaves. Such organ-specific distribution may follow well-recognized ecological adaptive principles: bitter secondary metabolites in leaves serve as chemical defenses against environmental stress, while sweet and umami-related compounds in fruits may facilitate animal-mediated seed dispersal [34,35]. These observations suggest a tentative association between bitter taste and predicted pharmacological potential, together with the preferential accumulation of sweet candidate bioactive metabolites in fruits.

The six dual-annotated metabolites are illustrated in Figure 7, providing chemical support to interpret our organ-resolved metabolomic results within broader phytochemical studies of medicinal plants. For instance, quercetin exhibits well-documented antioxidant and anti-inflammatory activities across multiple plant species [45]. Its high enrichment indicates that fruits represent a promising reservoir of flavonoid-related candidate compounds, beyond their predicted favorable sensory characteristics. As characteristic sesquiterpene lactones from *Arnica montana*, helenalin and dihydrohelenalin are reported to inhibit NF-κB-mediated inflammatory signaling pathways [46,47]. The prominent root-specific accumulation of helenalin provides tentative metabolomic evidence supporting the putative medicinal value of roots. Nevertheless, targeted compound confirmation and functional assays are essential to validate this hypothesis.

Conventional phytochemical research on *T. paniculatum* has largely been conducted as separate workflows of nutritional profiling, compound isolation, and pharmacological assessment [15,17,30,48]. By contrast, this study integrates untargeted organ-resolved metabolomics with multi-database functional annotation, providing an efficient framework for candidate metabolite prioritization and a chemical basis for differentiated organ utilization and functional food development. In line with standard practice in exploratory plant metabolomics, three biological replicates per organ were included [5,6,7]. Assessment of intra-group biological variability via Pearson correlation and PCA confirmed that inter-organ metabolic divergence was substantially greater than within-group variation, supporting the reliability of the observed organ-specific patterns. As is inherent to discovery-phase metabolomic studies, all annotations and functional associations remain putative and require further confirmation via authentic reference standards, targeted quantification, and experimental validation. Follow-up studies with larger sample cohorts will improve detection sensitivity for low-abundance metabolites, and combined approaches incorporating targeted quantification, sensory evaluation, and bioactivity assays will verify and extend the findings presented here.

## 5. Conclusions

Untargeted UPLC–MS/MS metabolomics enabled the presumptive annotation of 4521 metabolite features across four organs of *T. paniculatum* and revealed substantial organ-specific variation in phytochemical profiles. Matching these entries against the TCMSP database retrieved 348 metabolites with associated pharmacological records, of which 55 met the prioritization thresholds for predicted oral bioavailability and drug-likeness. These database annotations serve only for candidate prioritization and do not represent disease-specific activity or therapeutic efficacy of *T. paniculatum*. FlavorDB and MFood-Flavor Innovation provided database-derived flavor descriptors for candidate compounds, but these annotations do not establish actual sensory activity or perceptible taste in the plant. The observed organ-dependent relative abundance patterns generate testable hypotheses for subsequent targeted quantification and sensory evaluation, rather than constituting evidence of actual sweetness, bitterness, umami taste, or superior organ palatability. Six metabolites annotated with both pharmacological and flavor database entries carry concurrent putative bioactivity and database-assigned flavor attributes. In brief, these in silico profiling results provide a prioritized candidate library to guide follow-up validation studies, including targeted quantification, structural confirmation of metabolites, bioactivity assays, and formal sensory evaluation.

## Figures and Tables

**Figure 1:**
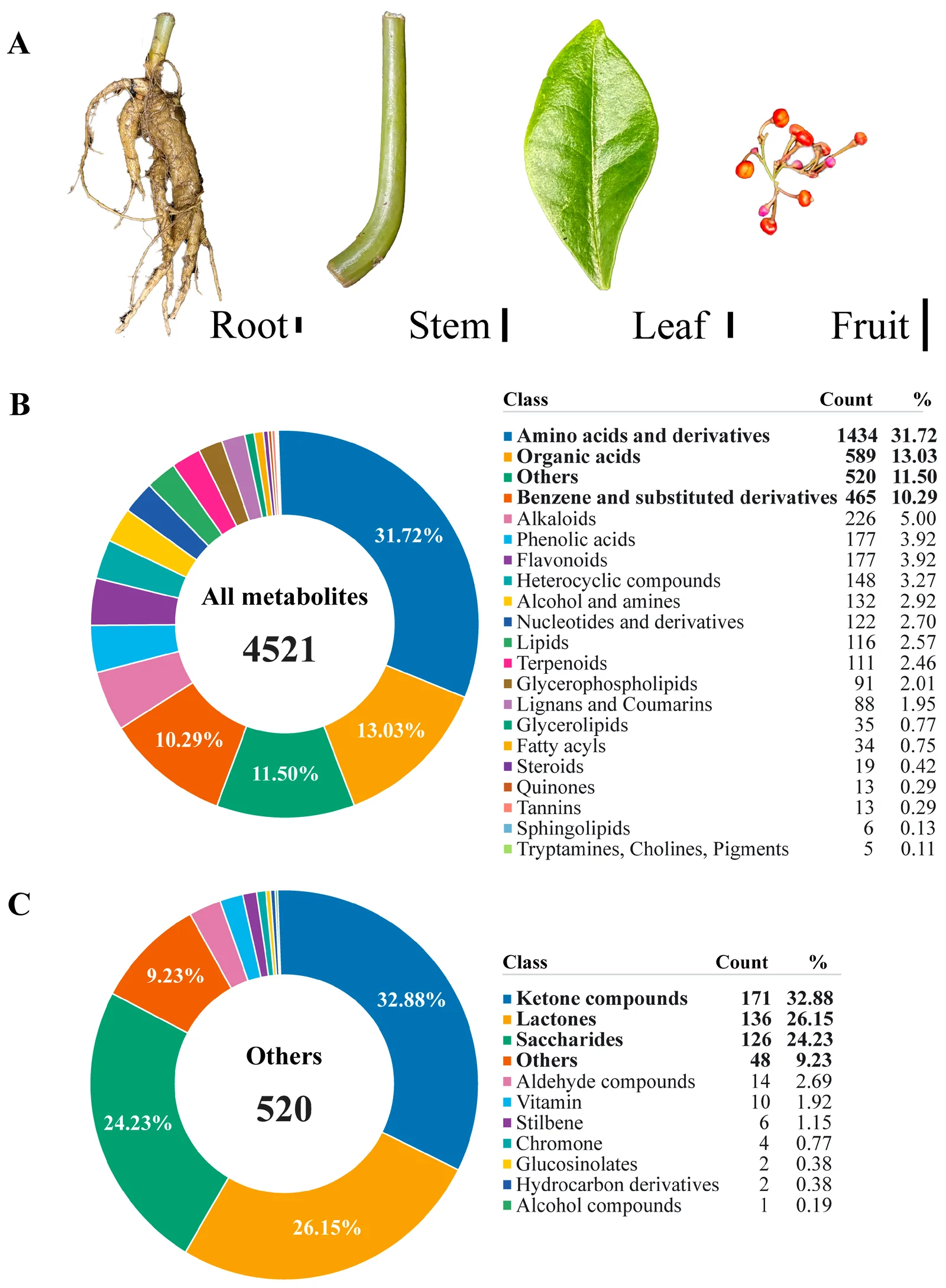
Metabolite profiles of four *T. paniculatum* organs. (**A**) Photographs of root, stem, fruit, and leaf. (**B**) Chemical classification of the 4521 putatively annotated metabolites. The four most abundant compound classes are highlighted in bold black font. (**C**) Subclassification of the 520 metabolites grouped under the “Others” category, mainly comprising ketones, lactones, and saccharides. Numbers represent metabolite counts, and percentages denote relative proportions within the corresponding dataset.

**Figure 2:**
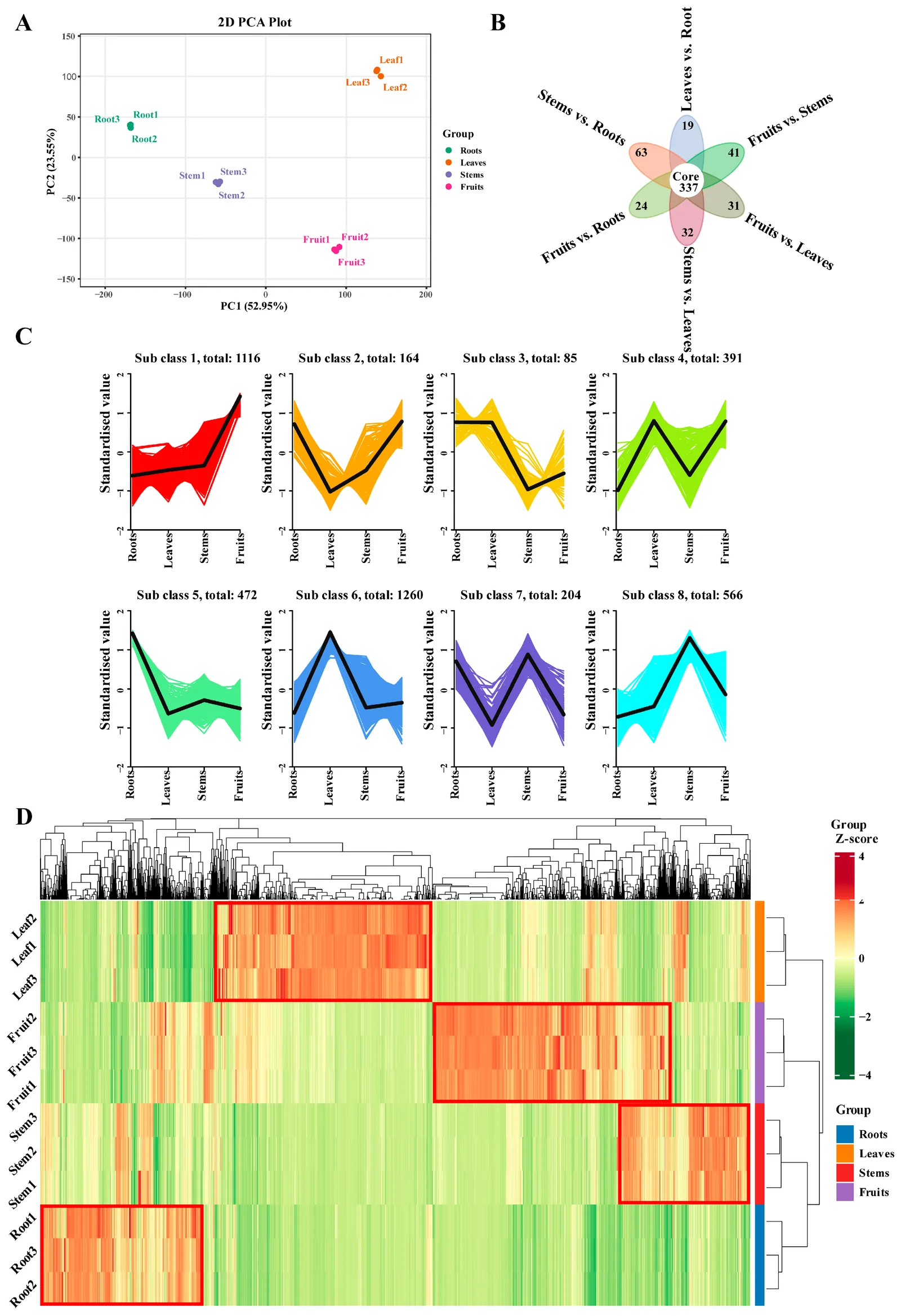
Metabolic variation in *T. paniculatum* organs. (**A**) PCA score plot for root, leaf, stem, and fruit. Colors represent four organs: green = roots, orange = leaves, blue = stems, magenta = fruits. (**B**) Overlap of differentially accumulated metabolites (DAMs) sets across the six pairwise organ comparisons. The core set contained 337 DAMs shared across all six comparisons. Numbers in the Venn diagram indicate shared or comparison-unique DAMs, not the total DAM count for each pairwise comparison. (**C**) K-means clustering of DAMs. (**D**) Hierarchical-clustering heatmap of 12 samples from four organs. The red boxes highlight metabolite clusters enriched in specific organs. The color bar indicates Z-score of standardized metabolite abundance, with red for high abundance and green for low abundance; side bar colors denote sample organ groups (blue: roots, orange: leaves, red: stems, purple: fruits).

**Figure 3:**
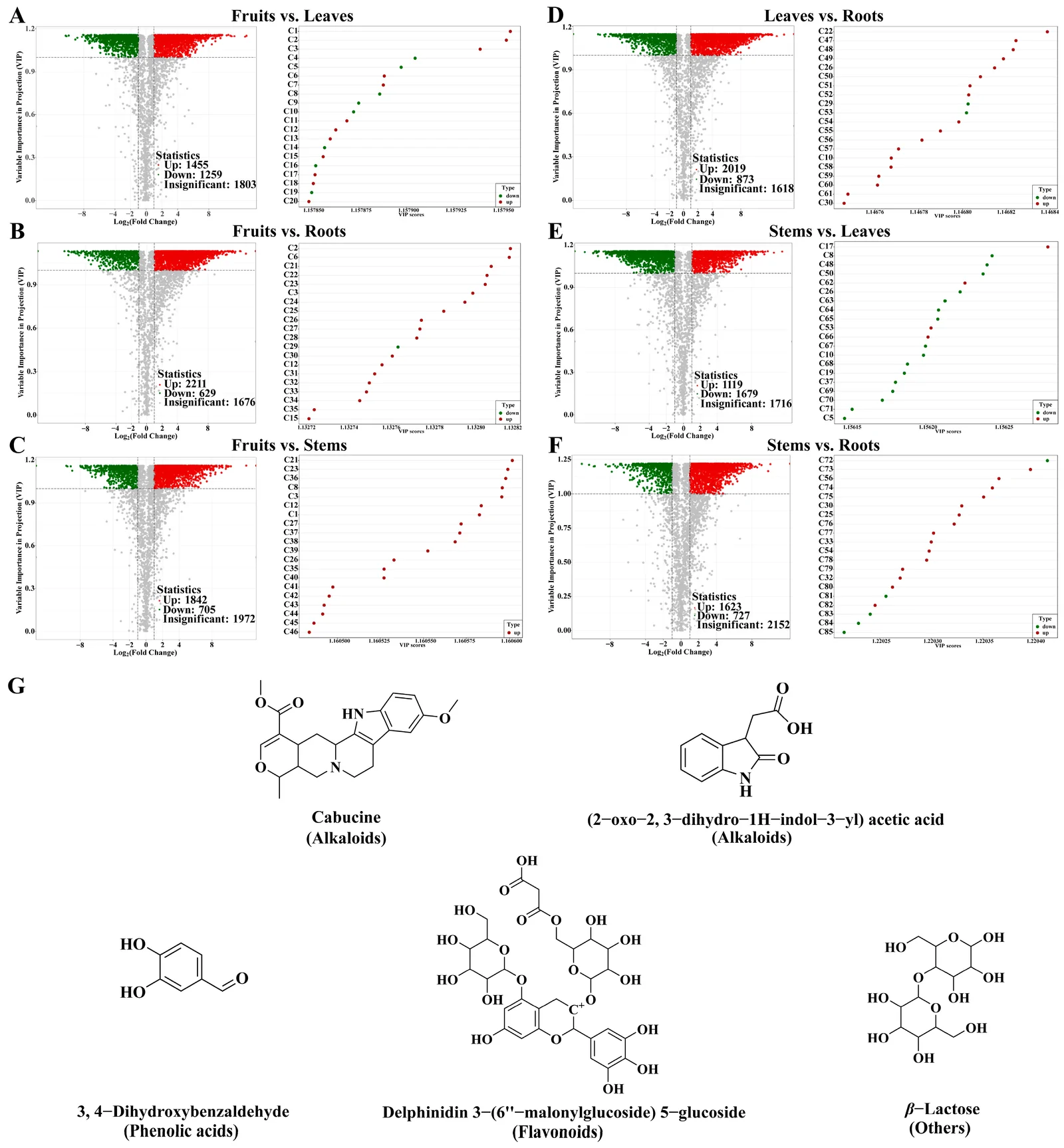
Pairwise comparisons of DAMs. (**A**–**F**) Volcano plots for each pairwise comparison with adjacent plots showing the top 20 DAMs ranked by VIP value. The full names of the corresponding compounds are provided in Table S2. (**G**) Chemical structures of five recurring DAMs detected in no fewer than three pairwise comparisons. *p*-values in volcano plots were calculated using a two-tailed Student’s *t*-test for pairwise organ comparisons.

**Figure 4:**
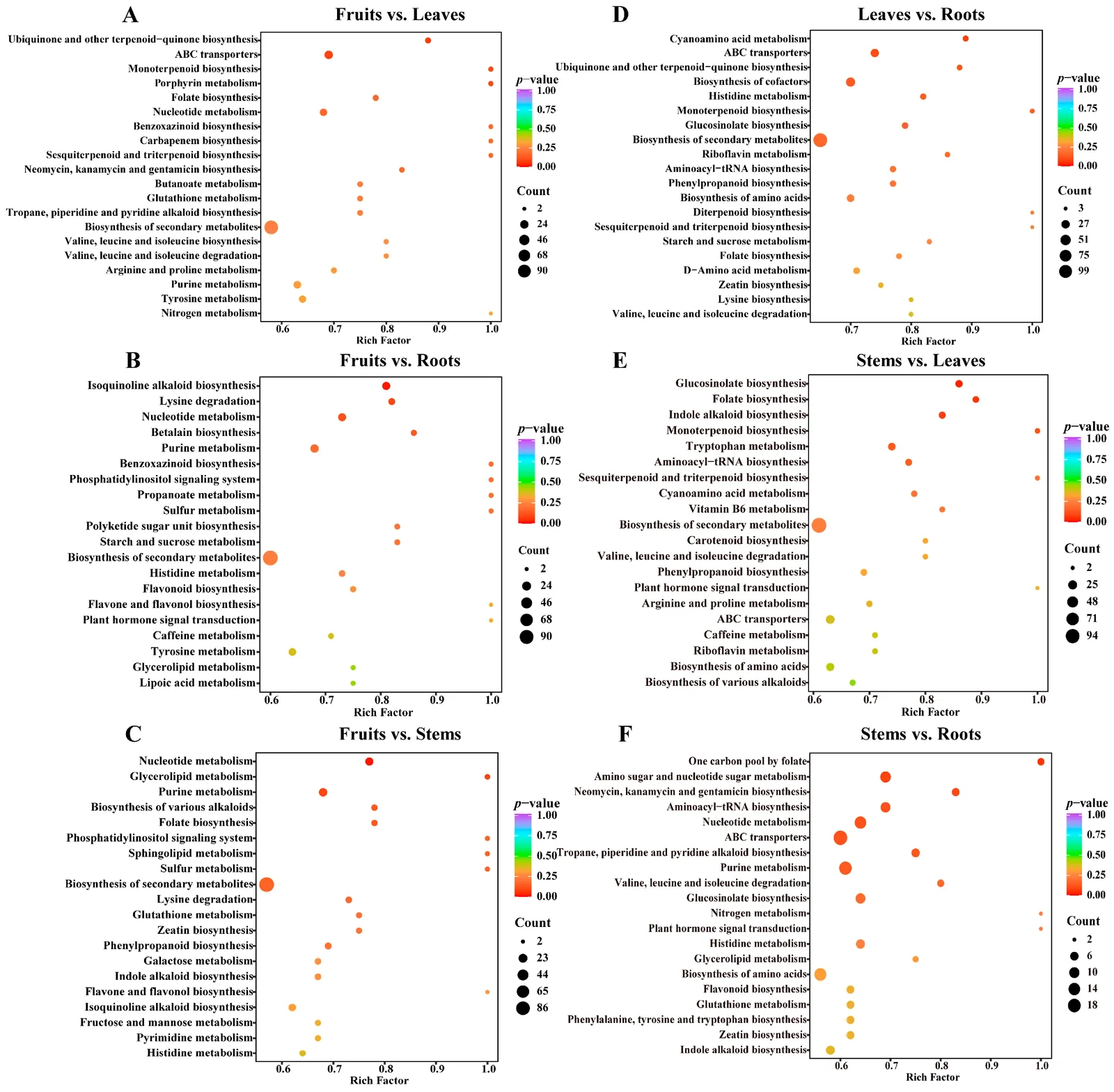
KEGG pathway enrichment analysis of differentially accumulated metabolites (DAMs) from six pairwise organ comparisons: (**A**) fruits versus leaves; (**B**) fruits versus roots; (**C**) fruits versus stems; (**D**) leaves versus roots; (**E**) stems versus leaves; and (**F**) stems versus roots. The 20 most significantly enriched pathways in each comparison are shown. The *x*-axis represents the rich factor. Dot color represents the *p*-value, with red indicating lower values and purple indicating higher values. Dot size represents the number of DAMs assigned to each pathway.

**Figure 5:**
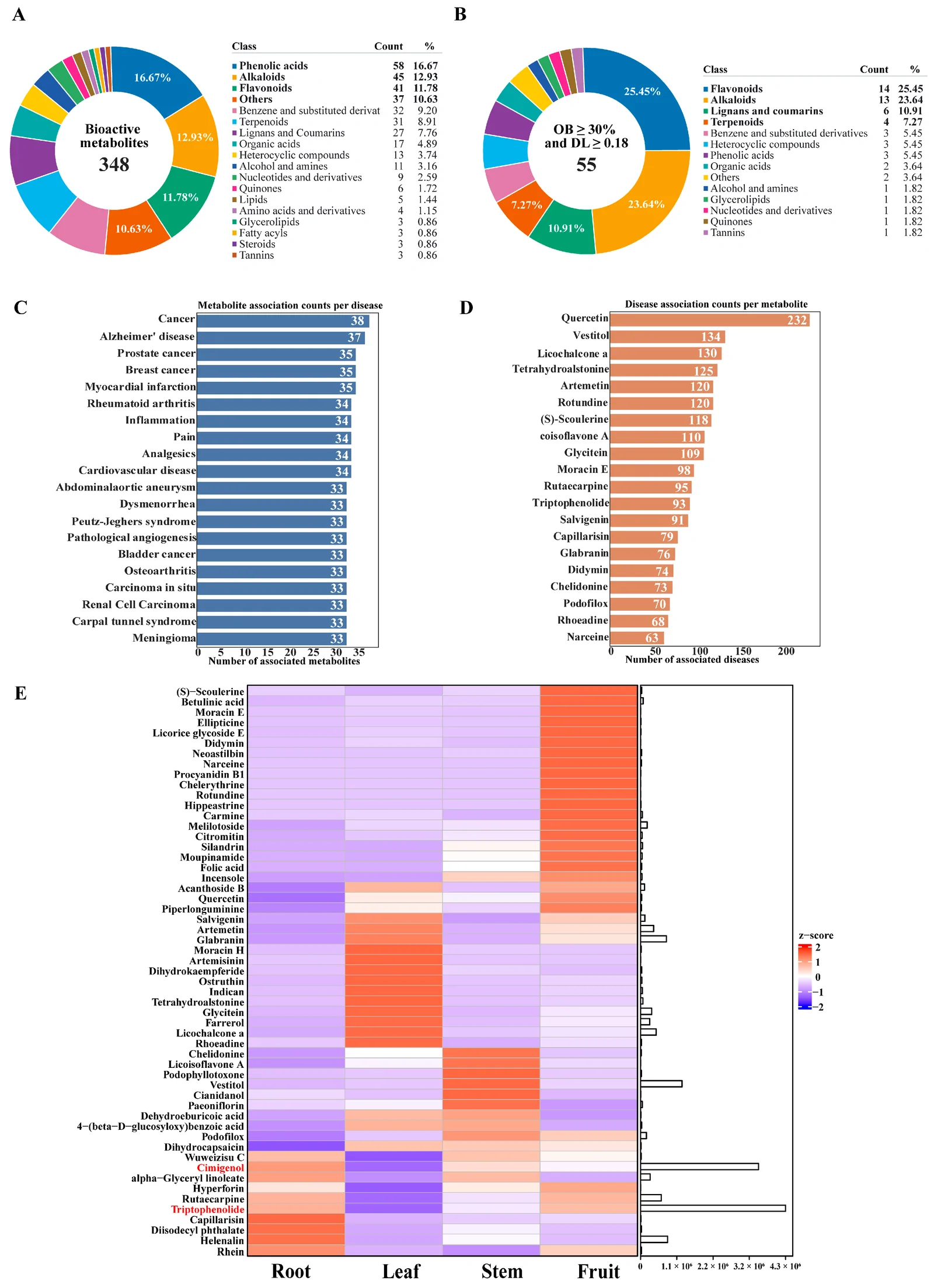
Summary statistics of TCMSP-screened candidate metabolites and their database-recorded disease associations. (**A**) Chemical classification of presumptively annotated metabolites with TCMSP pharmacological annotations. (**B**) Chemical classification of candidate metabolites satisfying the OB ≥ 30% and DL ≥ 0.18 prioritization thresholds. (**C**) Top 20 disease terms ranked by the number of associated candidate metabolites. Counts represent database-record frequencies only and do not imply disease-specific activity, therapeutic efficacy, or clinical relevance of *T. paniculatum*. (**D**) Top 20 candidate metabolites ranked by the number of associated disease records. Counts reflect database-curated associations only, not the strength of pharmacological activity or therapeutic efficacy. (**E**) Heatmap showing normalized relative abundance of candidate metabolites across organs. Color gradient from blue (low) to red (high) indicates relative abundance levels. The adjacent bar plot summarizes total relative abundance across organs, with two representative candidate metabolites labeled in red.

**Figure 6:**
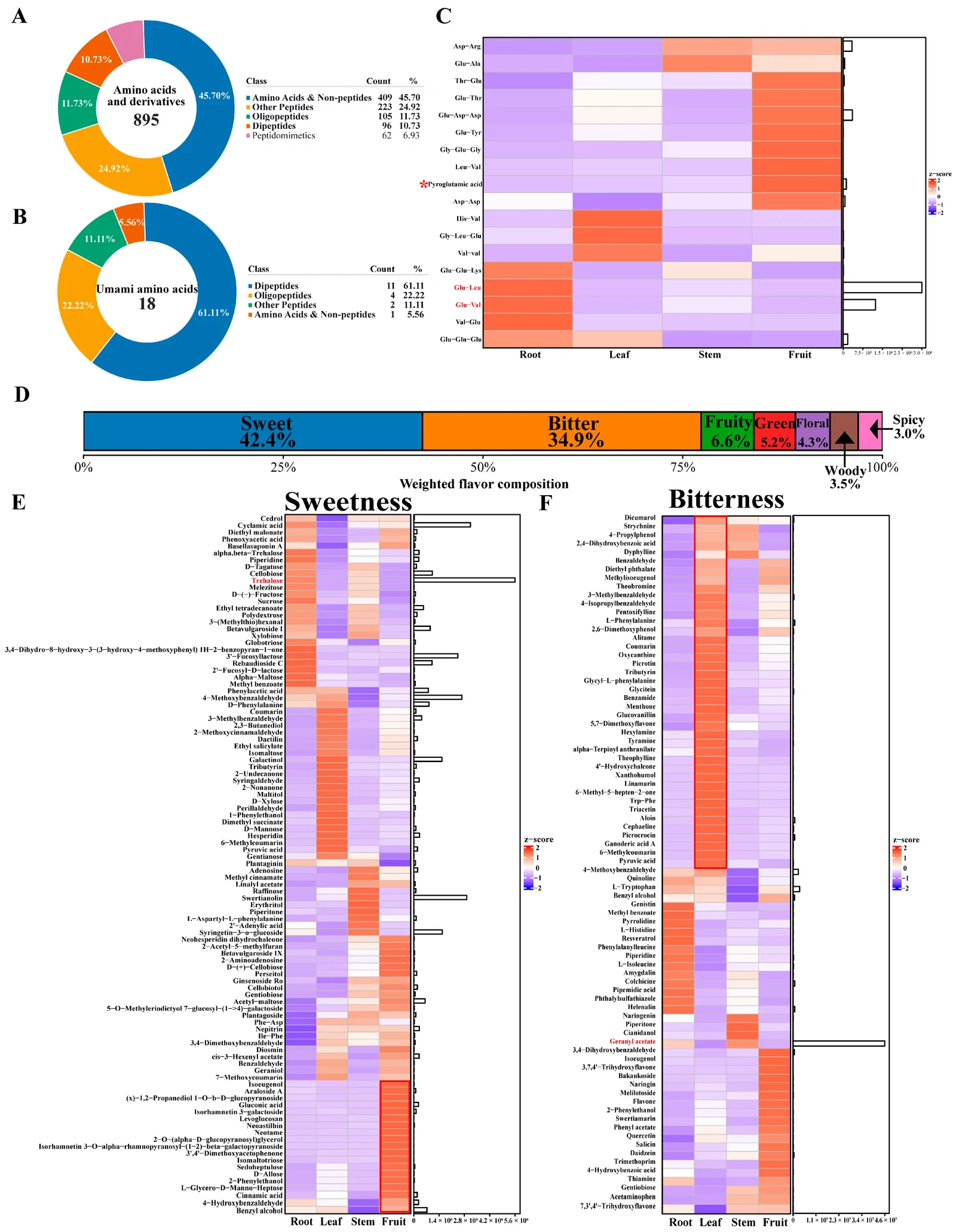
Profiling of candidate flavor-related metabolites. (**A**) Chemical classification of 895 amino acid-derived metabolites. (**B**) Classification of umami-related amino acid metabolites. (**C**) Heatmap showing the organ-dependent relative signal patterns of putatively annotated metabolite candidates assigned umami descriptors by MFood-Flavor Innovation. The red asterisk denotes pyroglutamic acid, the only metabolite bearing both MFFI umami annotation and TCMSP oral bioavailability (OB) and drug-likeness (DL) values, indicating predicted pharmacological relevance. These flavor descriptors are database-derived annotations and do not demonstrate perceptible umami taste or sensory activity in *T. paniculatum*. The color scale represents relative abundance patterns. Thr-Glu and Glu-Thr are presented as separate putative sequence-isomeric dipeptides. (**D**) Proportional statistics for metabolites annotated with seven flavor categories. (**E**,**F**) The heatmaps show the relative abundance of candidate sweet- and bitter-tasting metabolites. Blue corresponds to low abundance and red to high abundance. The bar plots on the right display the total metabolite abundance across the four organs. The highly abundant amino acid derivatives are highlighted in the red box.

**Figure 7:**
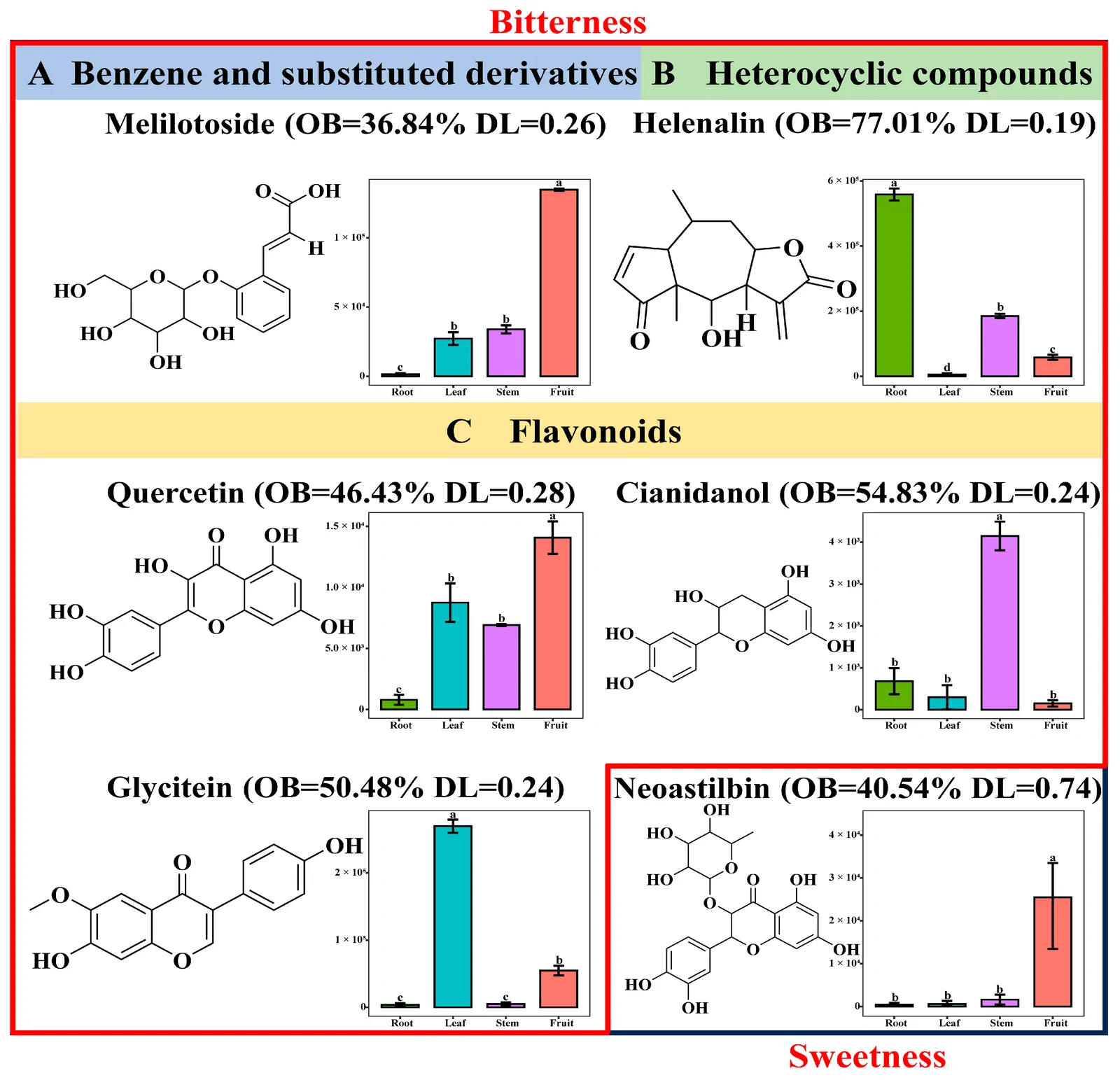
Organ-specific relative abundance of six candidate metabolites with both predicted bioactivity and database-annotated flavor attributes in *T. paniculatum*. (**A**) Benzene and substituted derivatives, represented by the bitterness-associated metabolite melilotoside (OB = 36.84%, DL = 0.26). (**B**) Heterocyclic compounds, represented by the bitterness-associated metabolite helenalin (OB = 77.01%, DL = 0.19). (**C**) Flavonoids, including the bitterness-associated metabolites quercetin (OB = 46.43%, DL = 0.28), cinamdanol (OB = 54.83%, DL = 0.24) and glycitein (OB = 50.48%, DL = 0.24), and the sweetness-associated metabolite neoastilbin (OB = 40.54%, DL = 0.74). Each panel shows the chemical structures and relative abundances of the corresponding metabolites in roots, leaves, stems and fruits. OB and DL values were obtained from TCMSP. Different letters indicate significant differences among the four organs (one-way ANOVA followed by Tukey’s HSD post-hoc test, *p* < 0.05). Bar colors correspond to plant organs: green = root, cyan = leaf, purple = stem, red = fruit.

## Data Availability

The datasets supporting the findings of this study are available in the Supplementary Materials. Any additional reasonable data inquiries may be directed to the corresponding author.

## Supplementary Materials

The following supporting information can be downloaded at https://www.mdpi.com/article/10.3390/metabo16100726/s1, Figure S1. Total ion chromatograms (TICs) of quality control (QC) samples. Pooled QC samples were injected periodically to monitor analytical stability. Overlaid TICs acquired in negative (A) and positive (B) ion modes showed consistent retention times and peak intensities, indicating good technical repeatability and data reliability. Figure S2. Pearson correlation analysis of QC samples. Pearson correlation analysis was performed to assess the reproducibility of QC samples in negative (A) and positive (B) ion modes. Figure S3. Distribution of coefficient of variation (CV) values across samples. Empirical cumulative distribution function curves of CV values for QC and organ samples in negative (A) and positive (B) ion modes. Dashed lines indicate CV thresholds of 0.3 and 0.5, and cumulative proportions of 75% and 85%. The high proportion of QC features with low CV values indicates good analytical stability. Figure S4. OPLS-DA permutation validation for the six pairwise organ comparisons. Each model was evaluated using 200 label permutations after log_2_ transformation and unit-variance scaling. (A) Fruits vs. leaves, (B) fruits vs. roots, (C) fruits vs. stems, (D) leaves vs. roots, (E) stems vs. leaves, and (F) stems vs. roots. R^2^Y and Q^2^ values were used to evaluate model fitness and predictive ability, respectively. Lower permutation-derived *p*-values indicate better model reliability. Figure S5. Principal component analysis (PCA)-based univariate statistical process control of QC samples. PC1 score control charts were used to monitor instrumental stability in (A) negative and (B) positive ion modes. Each point represents one injection in the analytical sequence, with QC samples shown in green and biological samples in black. Table S1. All presumptively annotated compounds in *T. paniculatum* organs. Table S2. Classification of significantly differentially metabolites between different origin samples. Table S3. Identification of the potential pharmacological ingredients based on the OB and DL values in TCMSP database. Table S4. Associations between potential bioactive metabolites (OB ≥ 30% and DL ≥ 0.18) and their related diseases based on the TCMSP database. Table S5. Detailed sub-classification criteria for amino acid and peptide-related metabolites based on the ClassyFire chemical taxonomy. Table S6. Comprehensive umami annotation of metabolites based on the Food-Flavor database. Table S7. Comprehensive flavor annotation of metabolites based on the FlavorDB database.
